# The development of a brain injury survivor patient and public involvement group by a brain injury survivor

**DOI:** 10.1371/journal.pone.0302763

**Published:** 2025-05-09

**Authors:** James Piercy, Colin Hamilton, Robert Runcie, Christi Deaton, Alexis J. Joannides

**Affiliations:** 1 NIHR Brain Injury Medtech Cooperative, University of Cambridge, Cambridge, United Kingdom; 2 Department of Physiotherapy, Cambridge University Hospitals NHS Foundation Trust, Cambridge, United Kingdom; 3 Primary Care Unit, Department of Public Health & Primary Care, University of Cambridge, Cambridge, United Kingdom; Transilvania University of Brasov: Universitatea Transilvania din Brasov, ROMANIA

## Abstract

**Background:**

Patient and public involvement (PPI) in research is seen as key to ensuring applicability and impact. Undertaking PPI in people after brain injury has long been seen to be a challenge. In 2020 The NIHR Brain Injury MedTech Cooperative developed a programme with the aim of improving PPI involvement, impact and diversity in this population.

**Methods:**

Through a process of iterative development, a PPI programme was created. It built on an existing underutilised database of people after brain injury and their carers who were interested in engaging with PPI and utilised video-calling software. It was led by a Brain injury Survivor acting as Facilitator with admin support from the MedTech Cooperative.

**Results:**

To date 14 PPI sessions were completed supporting a total of 17 projects. The diversity of the panel members was comparable to that of the population at large. However, further work is needed, especially in engaging people experiencing homelessness, people living outside of England and those with communication impairments. Feedback from researchers was positive and specific impacts are stated.

**Conclusion:**

Through the leadership of a facilitator who has an understanding of the lived experience of brain injury a PPI programme has been developed. The use of a video-calling platform enabled a wider representation then a face-to-face group would have and techniques such as shortened sessions and single project presentations ensured engagement and impact.

## Background

Patient and public involvement (PPI) is acknowledged to be central to good research practice [1]. However, enacting it in practice is a challenge [1]. This can be particularly true in populations who may experience disability, with researchers finding it difficult to engage with these patients and their carers [1]. These challenges can lead to inequities in access to PPI opportunities.

Populations who have limited access or particular difficulties engaging with research are less likely to benefit from the improvements in care that research can bring [2]. Research is also likely to be less applicable and wasteful if rigorous PPI with disadvantaged groups is not conducted [1]. Due to the emphasis that funders now place on PPI, research projects without robust PPI with relevant groups may find accessing funding more challenging, leading to further health inequalities in such populations.

Brain injury is a leading cause of death and disability worldwide [3]. With sequelae including physical impairments, cognitive deficits, and communication challenges [4] brain injury survivors face many barriers to actively participating in PPI. With this increase in disability family often take on a caring roll for the survivor which may limit the ability for their family and carers to engage [5]. Particular challenges in research have also been identified which could be improved through the use of effective PPI. Recruitment challenges and retention during studies are significant barriers to brain injury research [6]. While the research into the effects of PPI into retention is in its early stages, there is good evidence into the positive effects of PPI on recruitment to studies. This is particularly true with involvement for those with lived experience [7]. Therefore, finding effective methods of engaging with and facilitating research involvement among brain injury survivors are key to ensuring that research is relevant to this population [6].

The NIHR Brain Injury MedTech Co-operative (Brain Injury MIC) works with patients, carers, NHS, charities, academia, inventors, small and medium sized enterprises (SME) and potential investors to support the development of new medical devices and healthcare technologies improving the effectiveness and quality of healthcare services. Since its inception the Brain Injury MIC has established a patient advisory group (PAG). This group is formed of those who have suffered a brain injury and carers of people who have had a brain injury. The PAG provides a governance role to the Brain Injury MIC as well as contributing to strategic direction. It has been key in a push to develop an effective PPI strategy. In this paper we describe the development of the Brain Injury MIC’s PPI programme, initiated by Brain Injury Survivors, led by a Brain Injury Survivor and contributed to by Brain Injury Survivors.

## Previous PPI offering and identification of gap

Until 2020 PPI in research supported by the Brain Injury MIC was dealt with by the individual research teams with only informal help from the MIC administrative team. This support entailed advertising projects to members of the Register for Healthcare Involvement and Technology Evaluation (RHITE) database (www.brainmic.nihr.ac.uk/RHITE). RHITE was developed in 2012 by a previous version of the Brain Injury MIC and is a database of people with brain injury and their families/cares who were potentially interested in supporting research. While the RHITE could be a means of connecting researchers to interested parties it was underused.

Following discussion, the PAG and Brain Injury MIC administration agreed that this under-use of the RHITE resource was making it difficult to achieve meaningful and timely patient involvement in line with the UK standards for Public Involvement [8]. A more focussed support programme for PPI was needed, especially to assist researchers at application stage to improve their research questions in discussion with brain injury survivors and to demonstrate early commitment to PPI.

An outreach programme was proposed by the Brain Injury MIC with support of the PAG to make connections between those with lived experience of brain injury on the RHITE database and research groups developing new interventions on the acquired brain injury pathway.

### Aims

The aims of the outreach programme were:

- To increase involvement of brain injury survivors and their family or carers in the development of brain injury research
- To widen the diversity of involved members to be more representative of the population as a whole. This to include but not be limited to geography, gender, age, ethnic group and educational background.

### Iterative development

The outreach programme was developed through a process of discussion and consensus building with the Brain Injury MIC and the PAG. It was led by a brain injury survivor active in public engagement who took the role of Facilitator. Administrative and financial support was provided by the Brain injury MIC. The Outreach Programme adopted a model of iterative evaluation and review after initial launch and subsequent sessions (Fig S1).

The initial meeting between the Brain Injury MIC and the Facilitator was undertaken in Nov 2020 to set expectations for the programme and format. Due to the Covid-19 pandemic the meeting participants agreed that an online platform was needed to protect patients, carers and researchers. The participants also discussed their expectations of what the programme in the first year should deliver:

- A series of online events suitable for patients and carers, in which they can hear about research being supported by the Brain Injury MIC and discuss with the principal investigator.
- A supporting document for people joining the sessions, with links to the Brain Injury MIC, its research programmes and ways to get involved in research.
- Three outreach sessions within 6 months
- A review of the outreach programme after the first three sessions

#### Pilot stage: (Month 0–3).

A survey was sent to the investigators of all supported research projects to gather information about the researchers’ requirements for involvement and participation of patients, carers and the public in their projects, for example, review of documents or serving on an advisory committee (S2 Appendix 1). Each group was asked to provide a lay summary of their project. Six completed forms were initially received and from these 3 were chosen to be featured in the outreach session.

A pilot event was set up to test the effectiveness of the model on attendance rates of PPI Panel Members and impacts on projects. It was run using the GoTo Meeting™ web platform. Potential Panel Members were identified through the RHITE database and by contact with the local Headway (www.headway.org.uk) group which offers support and signposting for brain injury survivors and their families. Invitations were sent to those registered on the RHITE database and through Headway to its contact list. The event was set to run for 90 minutes and featured an introduction to the Brain Injury MIC followed by a presentation on the difference between engagement, involvement and participation.

Examples of current projects were discussed by the Brain Injury MIC to demonstrate the differences between engagement, involvement and participation as well as highlight opportunities to be engaged. Panel Members were invited to register their interest in specific projects by contacting the programme manager or to register a more general interest in research by joining the RHITE database.

In line with best practice and following the guidelines from the Centre for Engagement and Dissemination [9] participants were offered reimbursement in the form of a £25 voucher. Time was allowed for questions and comments, with participants invited to share their own experiences of involvement in research. After the session all delegates were sent a copy of the outreach brochure (S3 Appendix 2) and reminded of the links to join RHITE. Contact information was provided to give feedback and ask further questions.

The effectiveness of the pilot stage was measured in simple metrics. THe numbers of projects featured and public participants recruited.

#### Stage 1: (Month 4–7).

Following the pilot session, a team discussion took place incorporating comments from Panel Members and reflection from the Facilitator. The following changes were made:

- Reduced duration
- Switch to Zoom. The platform was felt to be more versatile, and attendees were more familiar with using it.
- Shorter introduction. The overview of the Brain Injury MIC activity was reduced to a very short introduction.
- Less time given to education on the distinctions between participation, involvement and engagement.
- Guest speaker. An aim was set to invite a researcher to each outreach session to speak personally about their project.
- Improved web visibility. The Brain Injury MIC website was updated to include information about the programme, engagement, involvement and participation and how to access the RHITE database.

Three research projects were featured in each of the 4 online workshops in stage 1. One of these was given prominence with the inclusion of a presentation from the research team, but due to time constraints the other two were presented by the Facilitator. The researchers could ask the group direct questions about their research question, methodology and recruitment strategy. With consent from the Panel members, sessions were recorded and shared with the research team after the event.

#### Stage 2: (Month 8–20).

Feedback from researchers and Panel Members indicated that whilst featuring a number of research projects allowed for variety and wider interest, a more focussed approach might yield greater impact. Delegates told the team that duration should be limited to 1 hour and that morning slots were easier to handle. Delegates emphasised that the cognitive fatigue experienced by many brain injury survivors made sustained attention difficult.

In the most recent iteration, a single project was featured presented by a member of the research team. The sessions were facilitated by the Facilitator. This role was key in moving discussions on when needed and keeping conversations related to the topic under discussion. An active chairing role was found to be essential to guide the sessions and a voice independent of the research team was valuable. Key questions and issues which could be informed by the lived experience of brain injury survivors and their carers were agreed before the focus groups and time was allotted to cover these.

Greater attention was paid to recruitment of PPI Panel Members with efforts made to match people with direct experience of the topic under discussion and to improve the geographic, gender, age and ethnic diversity of the group. As well as existing contacts the events were publicised through the People in Research website (https://www.peopleinresearch.org). Interested parties were invited to apply and their suitability explored with simple questions about the nature of their direct experience, location and ethnic background.

To gain an understanding of progress against the stated aims of this project (Increasing involvement in PPI and increasing the diversity of PPI Panel Members) numbers of Panel Members attending each session were recorded and a demographic questionnaire was sent out to gain an understanding of the makeup of the group. To assess impacts of the PPI group on each research study presented, feedback forms were issued to all investigators of projects that were supported by the outreach programme. The forms were based on the Centre for Research in Public Health and Community Care Guidance for Researchers: Feedback document [10].

## Results

Since the start of the PPI group 17 projects have been presented. This included 3 from Small and Medium Sized Enterprises (SME). Eight were featured in the initial outreach document and presented in stage 1, a further nine presented by researchers in the sessions held during stage 2 (including the 3 projects from SMEs). A timeline and attendance at the outreach sessions are presented in Table 1.

**Table 1:** Number of delegates attending each PPI session.

	Month since start of project	Delegates Registered	Delegates Attending
Pilot session	Month 3	16	11
Stage 1 sessions	Month 4	18	10
	Month 5	10	6
	Month 6	5	0
	Month 7	8	8
Stage 2 sessions	Month 11	15	13
	Month 12	9	9
	Month 13	19	12
	Month 14	9	7
	Month 16	27	22
	Month 17	14	9
	Month 18	11	11
	Month 18	13	13
	Month 20	10	7

We have observed variation in attendance but have been unable to isolate factors to account for this. The time of day and day of week seem not to have an effect but the topic under discussion may have influenced attendance

The demographic makeup of the overall group can be found in Table 2 with geographical location in Fig S2. Geographical data shows a concentration of members in the south of England and Wales with limited membership in the north of England and Scotland. There are no members in Northern Ireland.

**Table 2:** Demographics of the PPI group.

	Male						Female					Non-Binary		
Gender	31 (58.49%)						21 (39.62%)					1(1.89%)		
Ethnic Group	Asian or Asian British			Black, African, Caribbean, or Black British			Mixed or Multiple Ethnicity			White		Any Other Ethnic Group		
	4(7.55%)			15(28.30%)			1(1.89%)			31(58.49%)		2(3.77%)		
Decade Born	1950-1959			1960-1969			1970-1979			1980-1989		1990-1999		2000-2009
	10(19.23%)			11(21.15%)			11(21.15%)			2(3.85%)		17(32.69%)		1(1.92%)
Sexual Orientation	Heterosexual/straight			Gay/Lesbian			Bisexual			Self-described: Asexual		Self-described: Fluid		
	45(86.54%)			2(3.85%)			3(5.77%)			1(1.92%)		1(1.92%)		
Disability				Yes							No			
				38(73.08%)							14(26.92%)			
Experience of Brain Injury			None		Patient			Family Member or Friend					Caregiver	
			2(3.85%)		37(71.15%)			9(17.31%)					4(7.69%)	
Highest Level of Educational Attainment		No Formal Qualifications		Secondary Education		Further Education			Undergraduate Degree or equivalent			Higher degree or equivalent		
		3(5.66%)		0(0%)		11(20.75%)			27(50.94%)			12(22.64%)		

Out of the 17 projects supported only investigators from seven projects provided feedback when requested. Results can be found in Table 3. While numbers are small all projects found the group “Very Helpful” or “Quite Helpful” and all investigators that responded to the question (n = 6) would recommend the group to colleagues. Changes were reported in 4 projects. In each of these changes a theme was present around how the project or device interacted with the patient or user, emphasising usability and reducing burden. These changes were to both to design of device and research process. In two projects no changes were made with one discussing that concerns were raised by the PPI group about data collection, but further clarification was sought, and no changes were needed. While not a change, this may have strengthened the proposal and confirmed that the approach taken was acceptable. With regards to overall comments on the PPI group there was a central theme of feedback around the benefit of being able to easily access the group and the possibility of a longer-term partnership with members of the group which researchers seemed to value.

**Table 3:** Feedback from researchers.

	Very Helpful	Quite Helpful	Not Helpful	
Overall, how helpful were the comments from the PPI group?	4	2	0	
	Yes		No	
Has the review informed or influenced the project?	4		2	
	Yes	No	Not Sure	Missing
Would you recommend the group to colleagues?	5	0	0	2
	Comments			
Any changes made	‘The user interface..... was modified’ ‘we modified the patient requirements specification for our product, adding references to making the device as unintimidating as possible given that patients may be in a scary situation during use.’ ‘Helped to shape control group intervention. Pragmatics of intervention delivery. Shaped content of outcome measurement battery.’ We received useful feedback from patients and their loved ones during the workshop on their experience of the NPH pathway at CUH. Patients told us they would prefer to minimise hospital appointments so we adjusted our pathway to include a one-stop assessment clinic, saving time and transport costs.			
Reason for not making changes	‘The discussion was centred around the accessing of the personal information to create a dataset to derive a clinical prediction rule. The study applied for CAG approval and the PAG was approached to discuss how the data were to be collected, handled, shared and subsequently destroyed. Best practice approaches were proposed and the PAG was in agreement in how the study was designed in this respect.’ ‘offered correspondence with a patient but unfortunately we were not at a stage in the project to involve the individual at that time’			
Overall comments	‘We were very grateful to have been invited to present to the Brain MIC PPIE group.’ ‘Our major difficulties have been in accessing clinical colleagues to complete our project, mainly as a result of the huge pressures in the NHS, the pandemic but also due to changes in personnel. Through our research panels we are able to contact patients for PPI (and we have over the past year done this on a number of projects) but our issue remains engaging clinicians.’ ‘We discussed repeating this workshop format as our project progresses so we can continually get feedback from patients and their family members ‘ ‘We have continued involvement with some of the group participants to continually get their feedback on the project’s outputs. Some participants were invited to our project closing event where they could speak about their experiences in front of clinicians and researchers.’			

Comments were received on the facilitation of the session which helped with its development and improvement.

“We had a very positive experience in part because we were able to send private messages to the facilitator during the session to keep the conversation on track. The facilitator said they would adopt this for future sessions. This worked particularly well with it being a virtual format as it would be harder to communicate privately in person and not disrupt the conversation.”

## Discussion

The development of meaningful PPI is often stated as a challenge within research [11]. Various methods and models have been described in the literature [12] however this example emphasises the flexibility advocated by Buck et al. [13]. From the start the team adopted a process driven by the PPI Panel Members of iterative evaluation, reviewing the format and potential impact after each session to deliver the aims of the project. Overall, the programme was successful in the stated aims of facilitating a diverse group of people after brain injury to participate in impactful PPI activities. However, work remains to be done. Key points are presented below.

### Understanding the population

Brain Injury can cause a wide range of issues and understanding the challenges faced by survivors and their families or carers was key to the development of this project. From the initial idea the PAG, formed of people who have suffered brain injuries and their carers, has been key in ensuring the project was of importance to this population. This was further developed by the Facilitator who is a survivor of severe brain injury and whose direct experience helped inform this process and was key to the success.

### Ensuring impact of the sessions

The aim of the outreach project was to have real involvement from Panel Members, listening to their thoughts and responses to issues more than disseminating information about projects. It aimed to have useful input from lived experience at all stages of the research cycle. Informing pre-application strategy, focusing research questions and modifying delivery of research projects which fit well with the NIHR UK Standards for Public Involvement [7]. The importance of being able to meaningfully input on research projects has been described before [11] and is seen to be a key element of PPI. Through the iterative development the Panel moved from aiming to influence a larger number of projects with less input from researchers to intensely inputting on one in each session. This enabled active input rather than a light touch approach, which the group found to be key in ensuring impact.

The role of an active and experienced Facilitator was found to be vital in ensuring that messages were understood and that the purpose of the session was clear to participants. Prior to each session the facilitator reviewed the researchers’ presentations to check for clarity, and in a pre-meeting, identified key questions or areas for discussion with the research team. The use of plain English was mandatory at all points. In the panel meeting the facilitator directed the questions and ensured Panel Members understanding and supported their participation to maximise impact.

### Maximising accessibility for people after brain injury using technology, structure and vouchers

By understanding the population, a focus on accessibility was acknowledged to be central in order to optimise participation. Simple ice breakers (Fig S3) were used to set the tone for the sessions. Keeping the event friendly and warm encouraged contributions form participants. While the choice of an online process was initially driven by the COVID pandemic, which made face-to face sessions impossible, the need to engage with vulnerable people meant that in-person group meetings were not feasible. The online format has worked well as a means of engaging patients and the public with research projects, allowing people from across the UK to take part in the process and, whilst there is still work to do, it appears to have widened the diversity of participants.

The use of the Zoom platform to host the events was driven by the familiarity of the system to users. The pandemic also meant that potential participants were likely to be familiar with the technology and able to engage without prolonged instruction. The widespread use of online platforms in PPI is a relatively new phenomenon [12] and the advantages and disadvantages are still being evaluated. It must be acknowledged, however, that there are groups that find accessing technology more difficult and by using this platform certain groups are may have been disadvantaged [14].

There are particular issues of importance for engaging people in PPIE after brain injury. Brain injury survivors commonly live with issues caused by cognitive fatigue and screen-based activity has been shown to be more likely to cause these issues [15], which can limit participation. In response to these concerns sessions have been time limited to 1 hour and following feedback from participants have been scheduled before lunch rather than in the afternoon when people are more likely to be tired.

The group format also means that people with severe communication impairment are more likely to find the sessions difficult to engage with [16]. The team is looking into offering bespoke sessions in these cases [17].

Researchers found the inbuilt ability in the Zoom platform to record and review the session at a later date valuable. This allowed follow up questions to be directed to participants and a good understanding of contentious areas in discussion. The use of ‘chat’ functions allowed participants to note questions as they think of them, rather than having to remember them and finding a time to raise them later, which in view of the high incidence of memory difficulties in this population [18] can be a particular challenge. The facilitators also found that these questions could be used to restart conversations or to redirect conversation which may have become fixed on a minor point.

It is well acknowledged that PPI group members should be reimbursed for their time and discussion [19]. Legislation around HMRC IR35 can make it problematic for public bodies to make direct payments to individuals so provision of vouchers for payment was selected, which has been found to be effective previously [19]. Contributors reported being happy with this model. Research programmes at application stage do not have funding to support PPI but the Research Design Service public involvement fund was able to cover the cost of these initial sessions.

### Ensuring representation from a representative group

Diversity in PPI is an acknowledged issue [20]. The project aimed to improve the geographic, ethnic and socio-economic diversity of the group and the final results showed some success. An initial challenge of the team was not having previous data on the diversity of the initial participants in the RHITE database. The group so far is England centric with a concentration in London and limited membership in the north of England, Scotland and non in Northern Ireland which does limit range of views.

Other factors, however the group was more representative of the country with 10% of the PPI group identifying as LGBT+ in comparison with 10.6% in the most recent census(Statistics, 2022) [21]. With 28% of the group describing themselves as Black, African, Caribbean, or Black British this group is a higher proportion then in the wider population (4%) [21].

One person stated that they were “homeless” which is often seen to be one of the most challenging populations to engage with PPI work [22]. People experiencing homelessness however are at significantly higher risk for ill health and brain injury with 53.1% suffering a traumatic brain injury across their lifetime [23] and expanding input from this population is likely to provide significant new insights for the group.

The wide ranges of presentations that people have after Brain Injury [4] also make ensuring appropriate representation difficult. At present no attempt is made to understand diversity of impairment within the group but is seen to be a key future development.

### Limitations

The authors acknowledge that the low response rate (41%) from research groups on the impact of the programme, means that the effectiveness of the programme cannot be fully known. Reminder requests were sent to participating group on 2 occasions after each outreach session. Limited time made it difficult to follow up beyond this point. The reasons for lack of response are unknown.

### Moving forward

The latest review of the programme has identified areas where the outreach session could be improved further.

- More effort needs to be made to improve the diversity of those attending the sessions. Identifying third sector organisations and support groups for communities of ABI survivors with specific types of injuries or conditions might be one way of doing this. In particular, geographical diversity will need to be further addressed.
- A related project to develop a patient portal is underway. This will improve public access to information about research projects, allowing greater transparency of the work of the Brain Injury MIC and act as a forum to recruit study participants and Panel Members.
- Currently the PPI group has been focused on early-stage projects. As the supported projects continue and move to stages such as dissemination the PPI group will have input and impact on these aspects.
- Further focussing on increasing awareness of the use of the PPI programme with SMEs and supporting them to develop solutions and products to address problems faced by Brain Injury Survivors.

**It should be made clear to research groups that feedback on the value of the session and impact on their project is an expectation of being part of the programme.**

## Conclusion

We have illustrated the development of an outreach programme, led by a Brain Injury Survivor that engages brain injury survivors and their families or carers in research. This programme was well attended and had impact on research in the field. Key learning from this programme includes ensuring measurement of diversity, supporting access through the use of online platforms, maximising impact through preparation of researchers ahead of the sessions, and a knowledgeable and empathetic facilitator.

## Supporting information

S1 Fig 1 (TIF)

S2 Appendix 1 (DOCX)

S3 Appendix 2 (DOCX)

S4 Fig 2 Geographical distribution of PPI group members by county. (TIF)

S5 Fig 3 Ice-breakers for online groups. (TIF)

## Abbreviations

ABI: Acquired Brain Injury
MIC: Med tech cooperative
PAG: Patient advisory group
PPI: Patient public involvement
PPIE: Patient public involvement and engagement
SME: Small and medium enterprise.
