# Supplementary figures and images for: The development of a brain injury survivor patient and public involvement group by a brain injury survivor
Source: PLoS One. 2025 May 9;20(5):e0302763. doi: 10.1371/journal.pone.0302763 (PMC12063848; doi:10.1371/journal.pone.0302763)

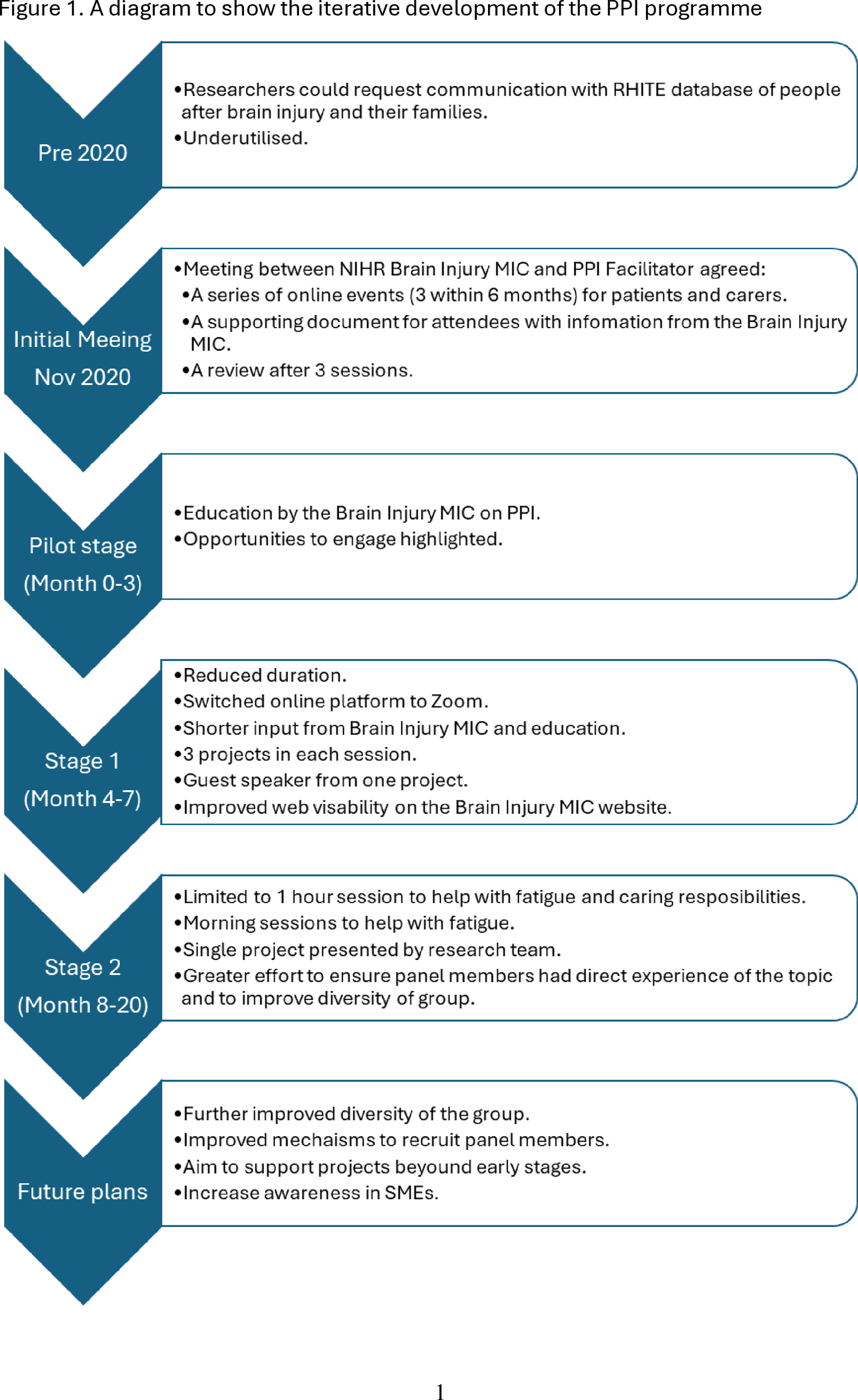

Supplement: S1 Fig 1 — (TIF) [file pone.0302763.s001.tif]

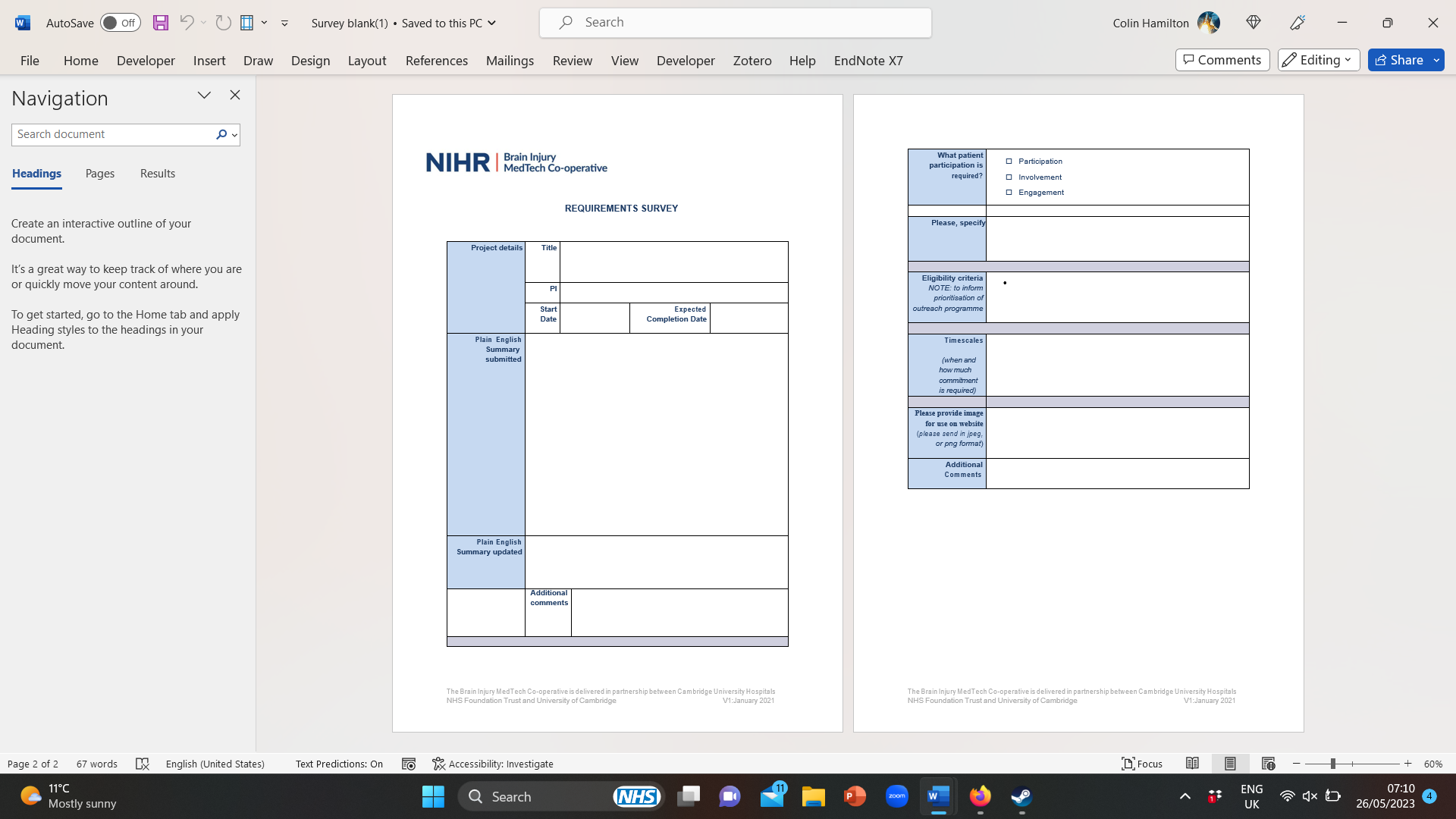
Appendix 1: Requirements Survey


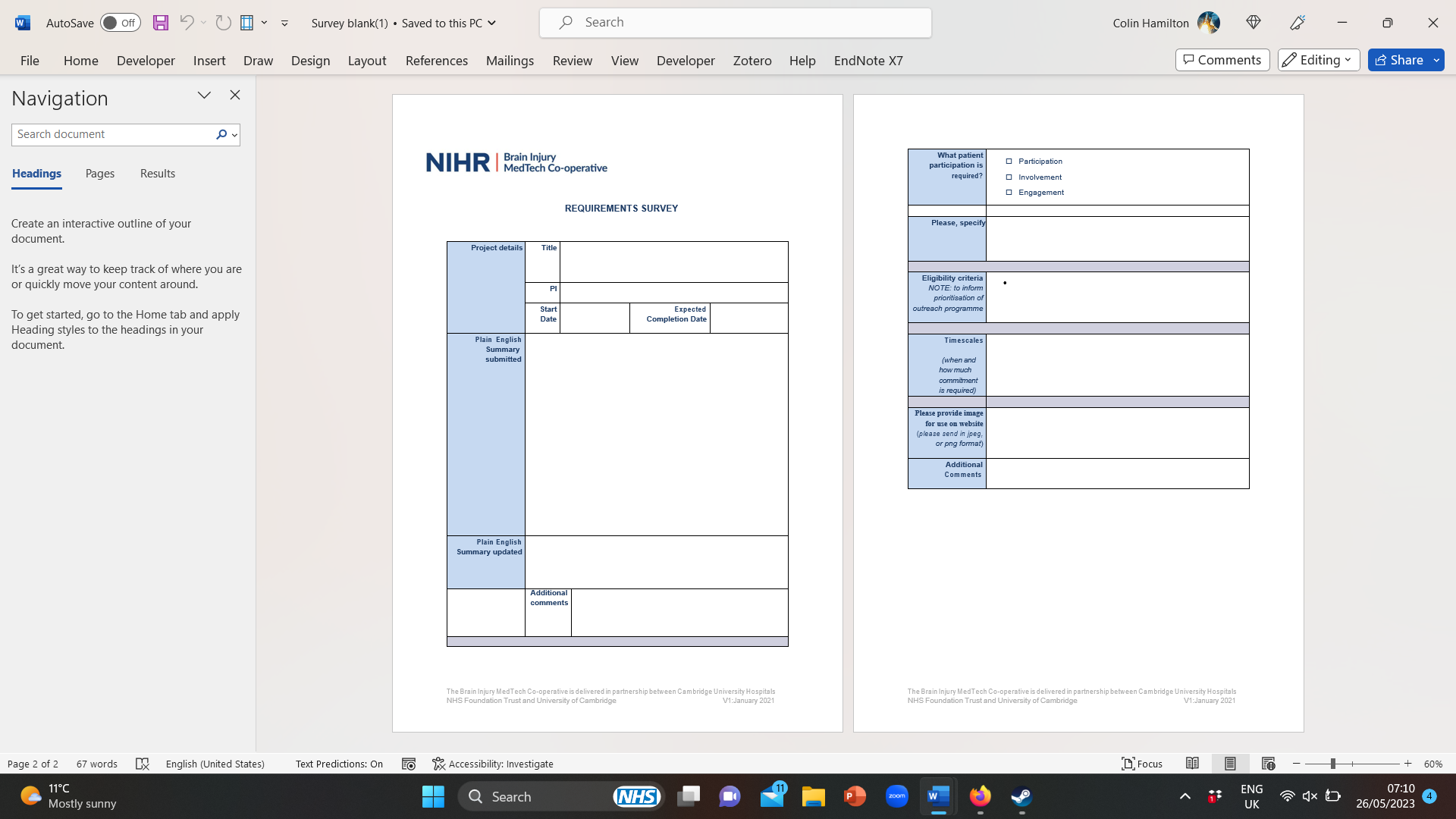

Supplement: S2 Appendix 1 — (DOCX) [file pone.0302763.s002.docx]

S3 Appendix 2 Outreach Document


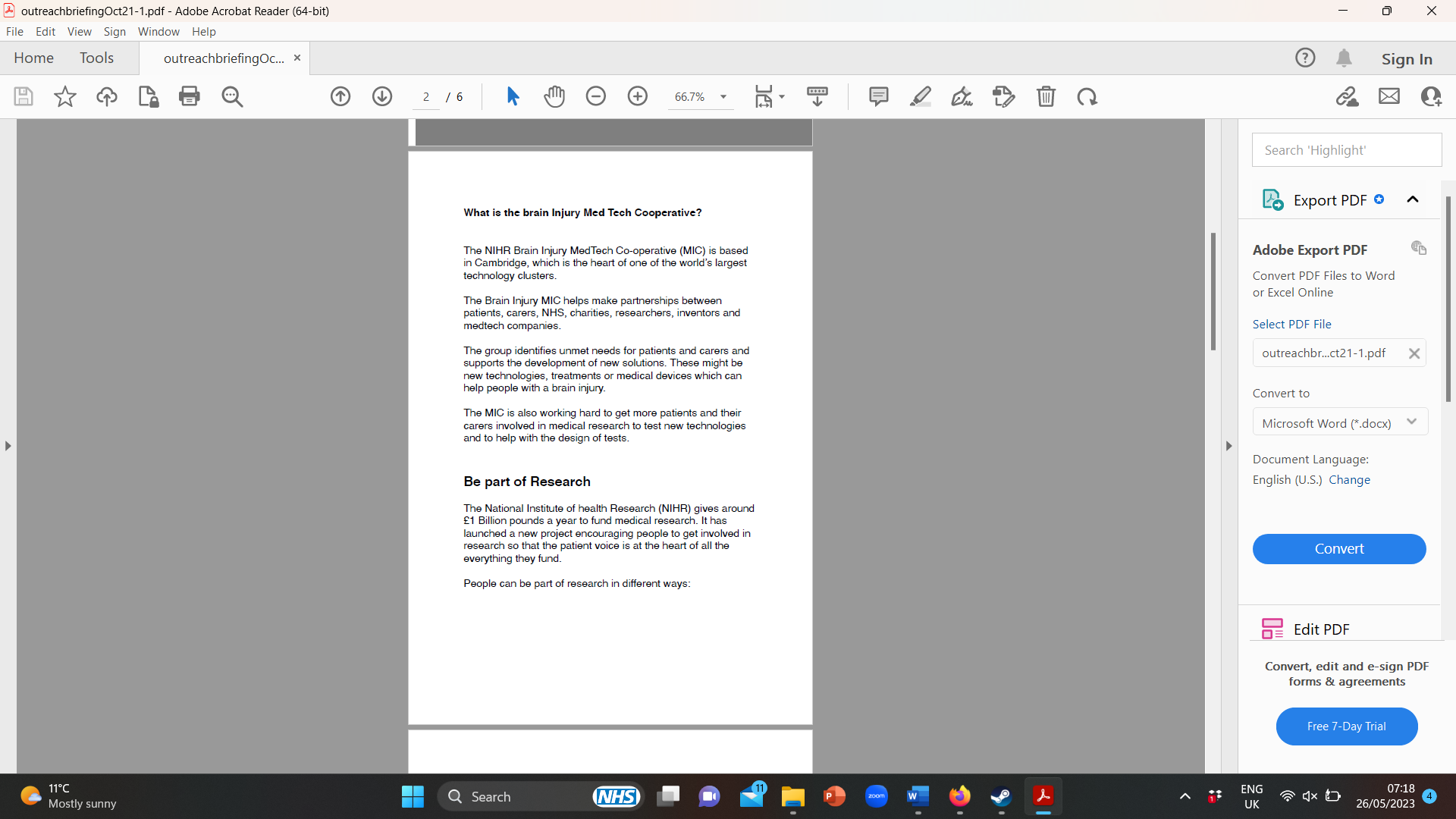


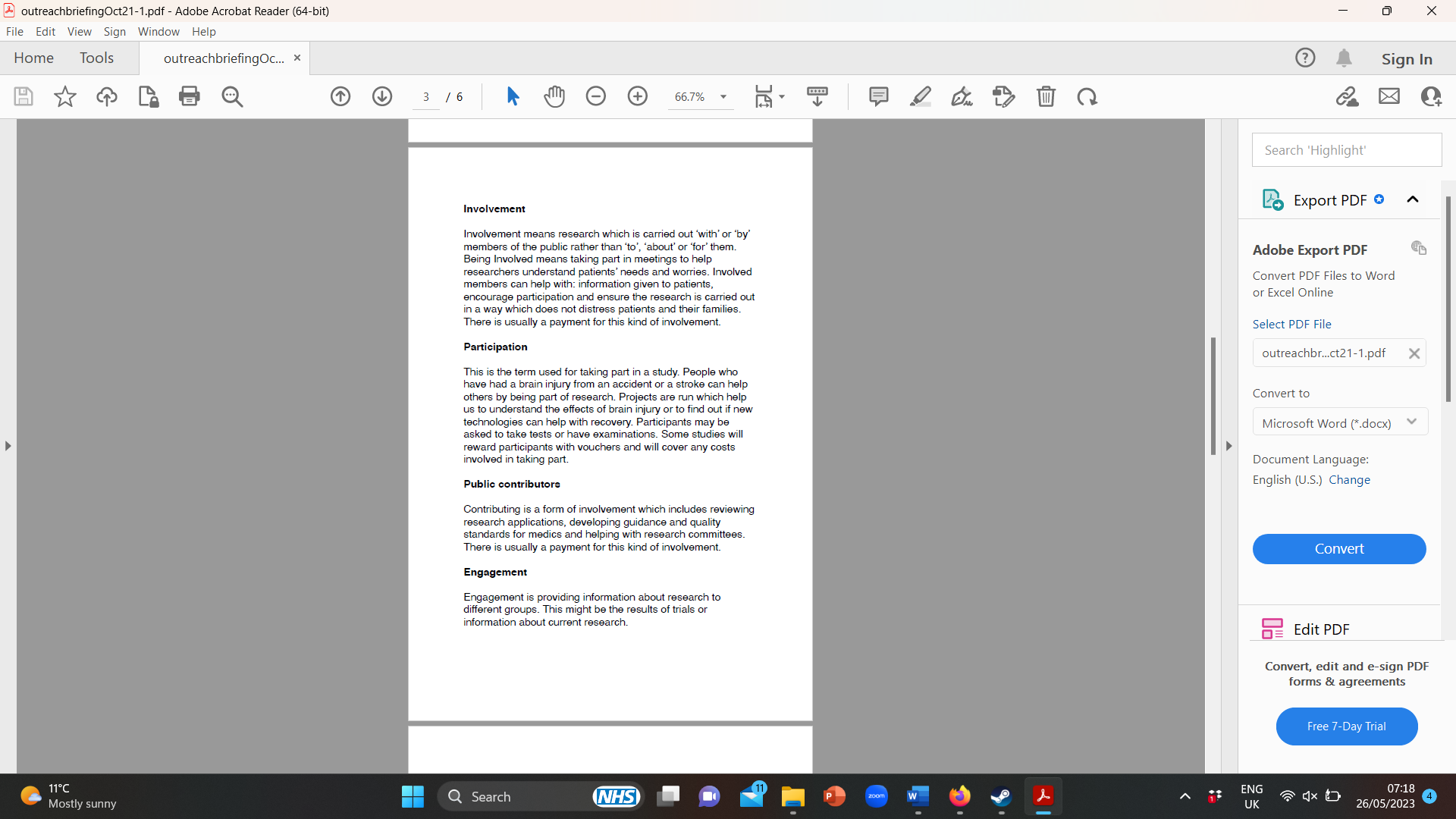


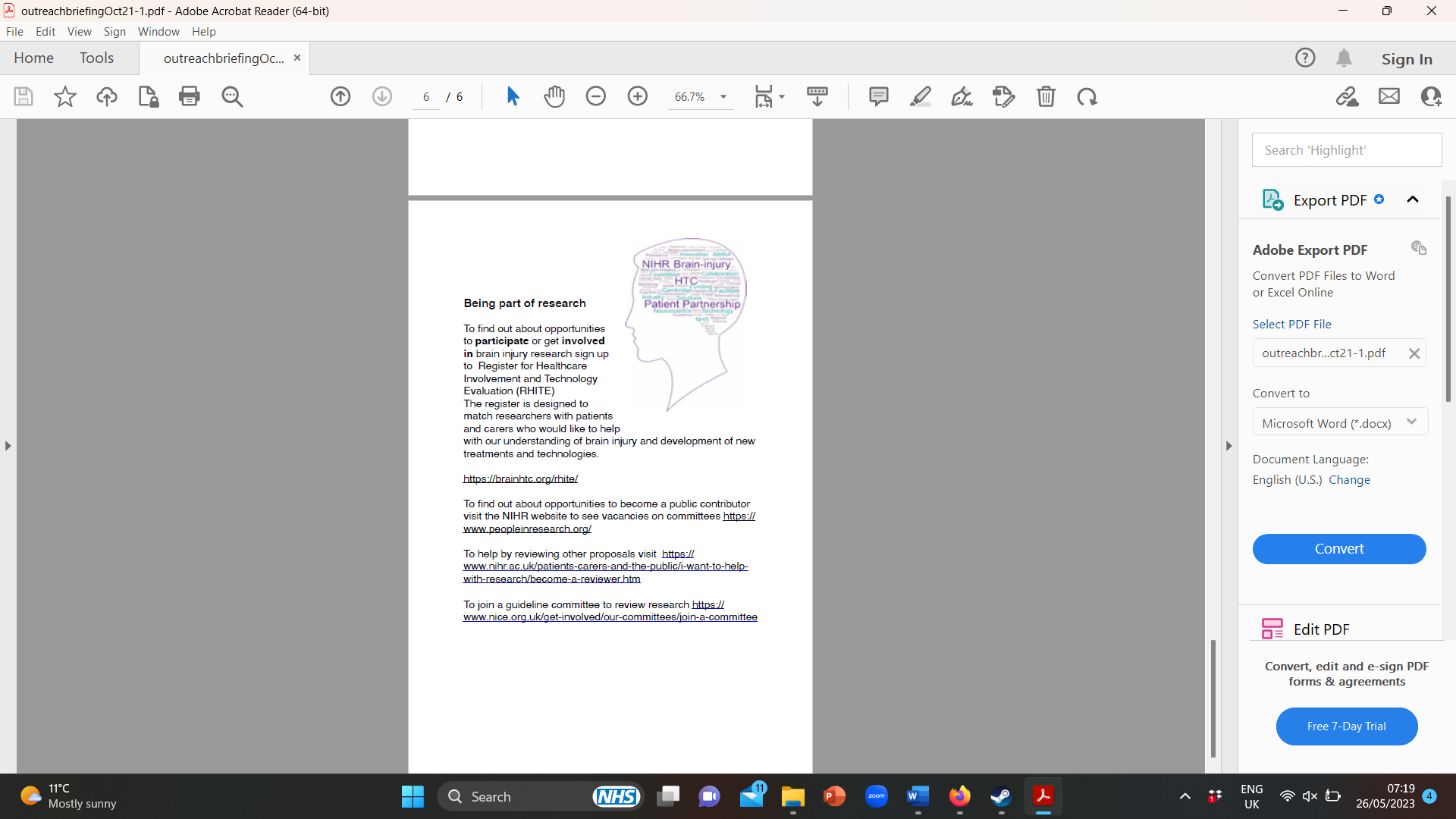

Supplement: S3 Appendix 2 — (DOCX) [file pone.0302763.s003.docx]

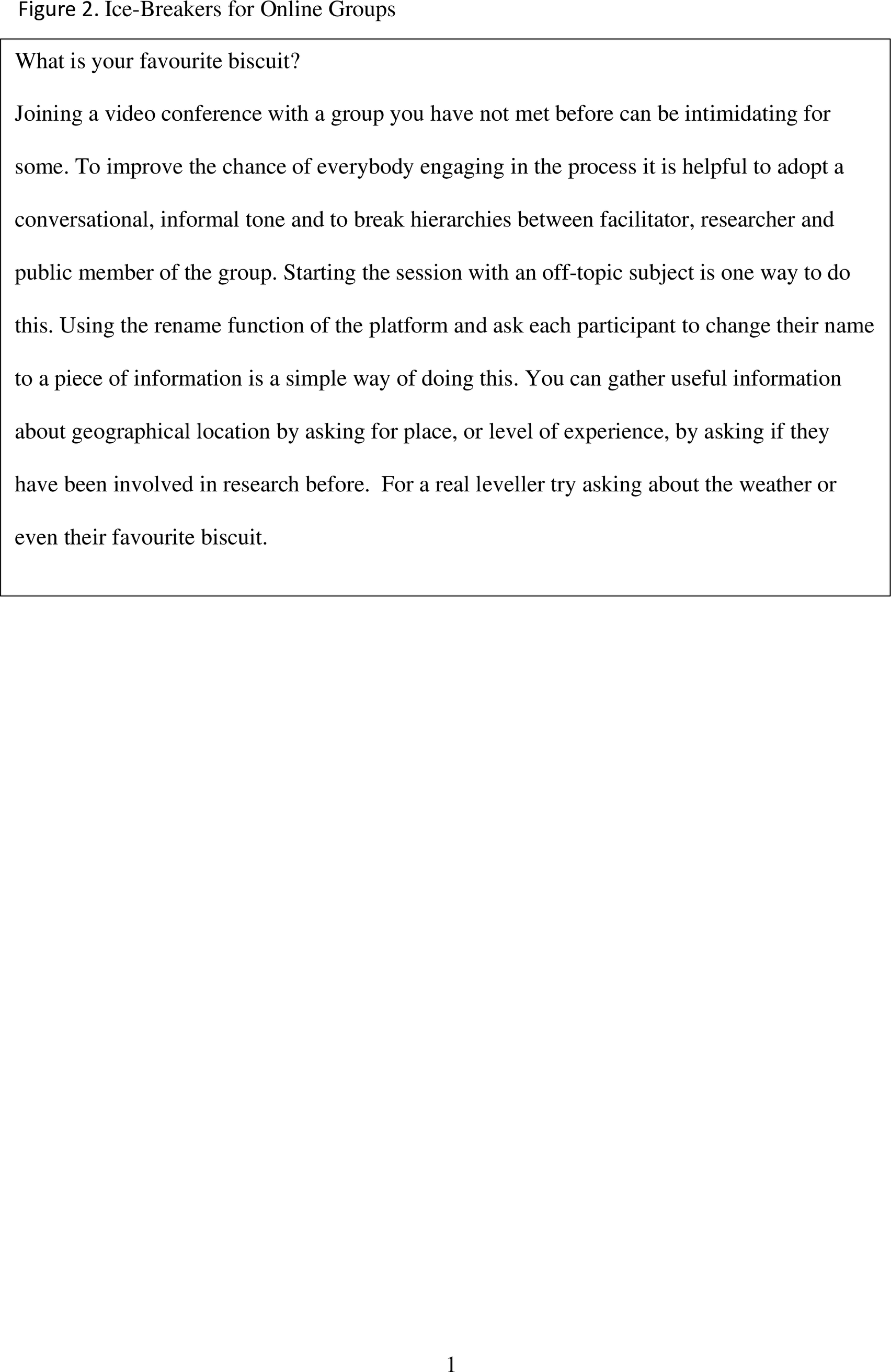

Supplement: S4 Fig 2 — (TIF) [file pone.0302763.s004.tif]

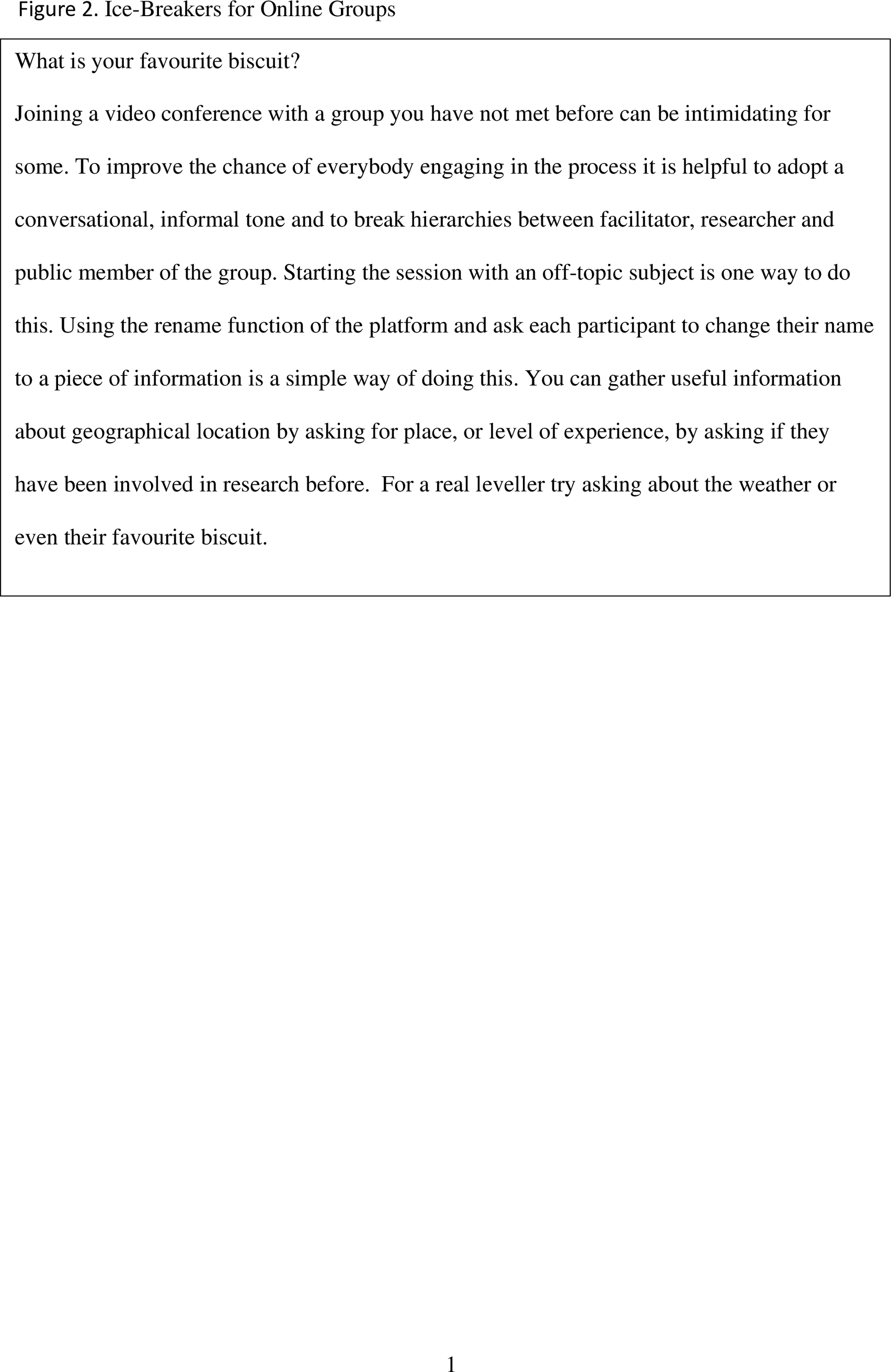

Supplement: S5 Fig 3 — (TIF) [file pone.0302763.s005.tif]
